# 3-D reconstruction of anterior mantle-field techniques in Hodgkin's disease survivors: doses to cardiac structures

**DOI:** 10.1186/1748-717X-1-10

**Published:** 2006-04-20

**Authors:** Dirk Vordermark, Ines Seufert, Franz Schwab, Oliver Kölbl, Margret Kung, Christiane Angermann, Michael Flentje

**Affiliations:** 1 Dept. of Radiation Oncology, University of Würzburg, Germany; 2 Dept. of Cardiology, University of Würzburg, Germany

## Abstract

**Background:**

The long-term dose-effect relationship for specific cardiac structures in mediastinal radiotherapy has rarely been investigated. As part of an interdisciplinary project, the 3-D dose distribution within the heart was reconstructed in all long-term Hodgkin's disease survivors (n = 55) treated with mediastinal radiotherapy between 1978 and 1985. For dose reconstruction, original techniques were transferred to the CT data sets of appropriate test patients, in whom left (LV) and right ventricle (RV), left (LA) and right atrium (RA) as well as right (RCA), left anterior descending (LAD) and left circumflex (LCX) coronary arteries were contoured. Dose-volume histograms (DVHs) were generated for these heart structures and results compared between techniques.

**Results:**

Predominant technique was an anterior mantle field (cobalt-60). 26 patients (47%) were treated with anterior mantle field alone (MF), 18 (33%) with anterior mantle field and monoaxial, bisegmental rotation boost (MF+ROT), 7 (13%) with anterior mantle field and dorsal boost (MF+DORS) and 4 (7%) with other techniques. Mean ± SD total mediastinal doses for MF+ROT (41.7 ± 3.5 Gy) and for MF+DORS (42.7 ± 7.4) were significantly higher than for MF (36.7 ± 5.2 Gy). DVH analysis documented relative overdosage to right heart structures with MF (median maximal dose to RV 129%, to RCA 127%) which was siginificantly reduced to 117% and 112%, respectively, in MF+ROT. Absolute doses in right heart structures, however, did not differ between techniques. Absolute LA doses were significantly higher in MF+ROT patients than in MF patients where large parts of LA were blocked. Median maximal doses for all techniques ranged between 48 and 52 Gy (RV), 44 and 46 Gy (LV), 47 and 49 Gy (RA), 38 and 45 Gy (LA), 46 and 50 Gy (RCA), 39 and 44 Gy (LAD) and 34 and 42 Gy (LCX).

**Conclusion:**

In patients irradiated with anterior mantle-field techniques, high doses to anterior heart portions were partly compensated by boost treatment from non-anterior angles. As the threshold doses for coronary artery disease, cardiomyopathy, pericarditis and valvular changes are assumed to be 30 to 40 Gy, cardiac toxicity must be anticipated in these patients. Thus, dose distributions in individual subjects should be correlated to the corresponding cardiovascular findings in these long-term survivors, e. g. by cardiovascular magnetic resonance imaging.

## Background

The risk of cardiac toxicity associated with mediastinal radiotherapy is well known. Multiple studies have addressed the prevalence of valvular disease, myocardial changes, coronary artery disease and the resulting risk of myocardial infarction or death from cardiac disease after thoracic radiotherapy, in particular after mantle-field irradiation in Hodgkin's disease [1-4]. Whereas the introduction of large radiation portals for concomitant irradiation of adjacent nodal sites by Kaplan [5] and implementation of effective multi-agent chemotherapy regimens by De Vita [6] led to a significant increase in cure rates in the 1970s and 1980s, clinical trials in the 1990s focussed on the reduction of radiotherapy doses and irradiated volumes. For instance, a multi-center trial of the German Hodgkin's Lymphoma Study Group (GHSG) established that in intermediate-risk patients cure rates are identical after extended-field and involved-field radiotherapy with 30 Gy, each following chemotherapy with COPP/ABVD [7]. In a subsequent series of trials, involved-field doses of 20 Gy and 30 Gy were compared in low-risk and intermediate-risk groups and early analyses in intermediate-risk patients suggest equivalence [8].

Despite these efforts to reduce radiation-induced late toxicity, of which heart disease is one aspect, oncologists and cardiologist are still seeing survivors of Hodgkin's disease treated in earlier decades, e. g. with extended-field radiotherapy of approximately 40 Gy, with or without chemotherapy.

Although some information is available on threshold radiation doses for certain cardiac toxicities such as coronary artery disease, pericarditis, or valvular changes, estimating that a critical dose range is between 30 and 40 Gy [9], a correlation of damage to particular cardiac structures and dose to the corresponding region has rarely been attempted, due to a lack of 3-D computed tomography data sets for patients reported in published series. Some authors have calculated the heart dose at a certain depth and used this value for statistical analysis [3].

In the present analysis, we reconstructed the dose to cardiac structures in patients treated with anterior mantle-field techniques, with or without boost, for Hodgkin's disease between 1978 and 1985. Dose-volume histograms were generated for heart cavities and coronary arteries by applying the information on original radiotherapy technique to CT data sets of test patients. The information thus obtained will form the basis of a detailed analysis of patterns of cardiac damage within an interdisciplinary project of cardiologists and radiation oncologists.

## Results

**Figure 1:**
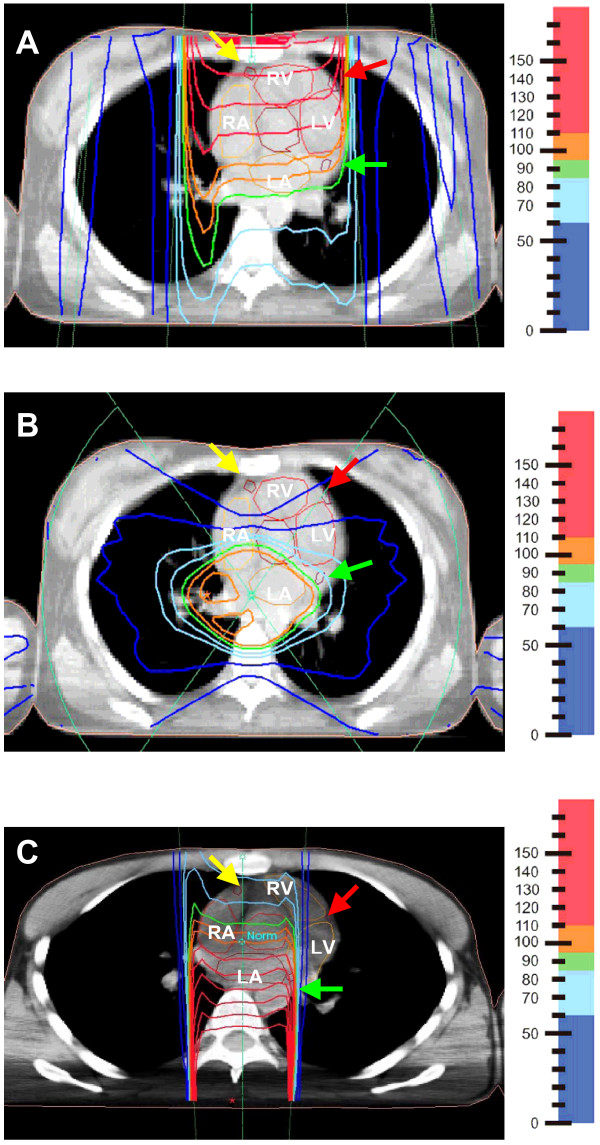
Reconstruction of dose distribution for typical cases of anterior mantle field (A), monoaxial, bisegmental rotation boost (B) and dorsal boost (C). Note the contours of heart cavities and position of coronary arteries (RA: right atrium, LA: left atrium, RV: right ventricle, LV: left ventricle, yellow arrow: right coronary artery, red arrow: left anterior descending (LAD) artery, green arrow: left circumflex (LCX) artery).

Of the 55 patients, 26 (47%) were treated with anterior mantle field (MF) alone, 18 (33%) with anterior mantle field and monoaxial, bisegmental rotation boost (MF+ROT), 7 (13%) with anterior mantle field and dorsal boost (MF+DORS) and 4 (7%) with other techniques, e. g. three-field technique (OTHER). Typical reconstructed dose distributions of anterior mantle field, rotation boost and dorsal boost are shown in Fig. 1. Anterior mantle fields were typically treated at 120 cm source-skin distance, single surface dose 1.3 Gy, single dose at prescription point (at approximately 7 cm depth) 2.0 Gy. For dorsal and rotation boost, the source-isocenter distance was 60 cm and typical single doses were 2 Gy at isocenter.

Mean ± SD total prescribed doses in the mid-third of the mediastinum were 36.7 ± 5.2 Gy for MF, 41.7 ± 3.5 Gy for MF+ROT (p = 0.003), 42.7 ± 7.4 for MF+DORS (p = 0.01) and 41 ± 2 Gy for OTHER. The mean contribution of boost doses to these total doses were 9.8 ± 2.2 Gy in MF+ROT and 10 ± 2.3 Gy in MF+DORS.

The results of a dose-volume histogram analysis of cardiac structures are shown in Table 1. The median values of individual minimal, maximal, mean and median doses were calculated for both atria and ventricles as well as for coronary arteries. Doses are given as relative values, to underscore overdosage to particular cardiac structures with specific techniques, and as absolute values in Gy, taking into account the prescribed total doses which differed between techniques. The absolute values represent an estimation of the dose the patients actually received in particular regions of the heart.

**Table 1:** Results of dose-volume histogram analysis. Results of dose-volume histogram (DVH) analysis of cardiac structures (RV: right ventricle, RA: right atrium, LV: left ventricle, LA: left atrium, RCA: right coronary artery, LAD: left anterior descending artery, LCX: left circumflex artery). Median (min-max) doses are given for patients treated with mantle field alone (MF), mantle field + rotation boost (MF+ROT) and mantle field + dorsal boost (MF+DORS), both as relative doses (in %) and absolute doses (in Gy). Values significantly different from MF are indicated by bold print (*= p < 0.05). Significantly higher relative doses to RV, RA and RCA with MF alone are compensated by reduced total prescription doses for MF technique and by sparing of right heart structures with boost techniques, resulting in comparable absolute total doses between techniques. (# indicates DVH parameters in which a significant inverse correlation between 2-D heart area shielded by block and the respective DVH value was observed.)

**structure**	**relative dose [%]**			**absolute dose [Gy]**		
**(# = significant correlation 2D–3D)**	**MF n = 26**	**MF+ROT n = 18**	**MF+DORS n = 7**	**MF n = 26**	**MF+ROT n = 18**	**MF+DORS n = 7**

**RV min #**	30 (16–98)	24 (12–49)	18 (14–87)	11 (3–35)	11 (5–22)	8 (6–40)
**RV max**	129 (108–147)	117 (98–141)*	114 (94–124)*	49 (23–58)	48 (43–58)	52 (39–52)
**RV median**	117(49–132)	101(64–118)*	94 (88–114)	43 (18–53)	43 (22–52)	43 (36–48)
**RV mean #**	114 (61–127)	99 (74–112)	85 (80–104)	41 (19–52)	41 (25–47)	37 (35–46)
**LV min**	18 (12–46)	16 (9–27)	15 (10–20)	7 (2–16)	8 (4–11)	6 (5–9)
**LV max**	119 (32–147)	107 (52–129)	96 (92–122)	46 (12–53)	45 (18–55)	44 (39–51)
**LV median #**	42 (17–99)	41 (19–95)	40 (22–55)	16 (6–41)	18 (7–39)	17 (10–25)
**LV mean #**	56 (17–91)	50 (26–83)	47 (33–58)	22 (6–35)	23 (10–34)	19 (15–25)
**LA min**	84 (28–97)	79 (48–95)	73 (40–89)	30 (10–37)	34 (16–40)	32 (18–37)
**LA max**	102 (84–116)	106 (86–124)	93 (85–110)	38 (18–46)	45 (36–49)*	43 (35–46)
**LA median**	92 (76–106)	98 (79–108)	80 (75–103)	35 (16–41)	41 (33–45)*	36 (33–43)
**LA mean**	92 (76–106)	97 (78–107)	81 (75–103)	35 (16–41)	41 (33–45)*	36 (33–43)
**RA min**	86 (20–103)	75 (37–104)	75 (43–101)	31 (8–38)	30 (17–44)	33 (19–42)
**RA max**	123 (100–142)	116 (99–143)	107 (91–119)*	47 (21–55)	49 (42–57)	49 (38–50)
**RA median**	105 (76–122)	101 (88–125)	90 (86–110)	39 (19–48)	43 (33–51)	41 (35–46)
**RA mean**	105 (68–122)	100 (84–122)	89 (86–110)	39 (19–47)	42 (32–50)	41 (35–46)
**RCA min**	101 (48–128)	90 (58–108)	84 (67–99)	37 (19–51)	38 (23–47)	38 (31–42)
**RCA max**	127 (99–147)	112 (93–144)*	109 (94–122)*	48 (22–56)	46 (40–56)	50 (39–51)
**RCA median**	120 (90–141)	104 (89–125)*	103 (92–120)	45 (22–55)	43 (37–55)	47 (38–50)
**RCA mean**	116 (91–136)	102 (86–121)*	96 (90–11,6)	44 (21–54)	42 (36–53)	44 (37–48)
**LAD min #**	23 (15–80)	20 (11–34)	18 (13–33)	9 (3–28)	10 (5–14)	8 (6–15)
**LAD max #**	109 (25–139)	104 (48–122)	92 (68–120)	39 (9–55)	44 (16–50)	40 (31–50)
**LAD median # **	60 (19–121)	66 (28–113)	39 (26–108)	20 (7–48)	30 (10–47)	18 (12–45)
**LAD mean # **	61 (19–112)	60 (30–96)	53 (35–81)	23 (7–46)	28 (10–39)	22 (16–34)
**LCX min #**	68 (22–93)	66 (38–91)	53 (28–79)	25 (8–37)	29 (13–38)	22 (13–33)
**LCX max**	91 (44–111)	96 (56–112)	87 (81–108)	34 (16–43)	42 (19–46)*	40 (33–45)
**LCX median #**	80 (22–95)	79 (42–95)	75 (40–95)	29 (8–39)	35 (14–40)	31 (18–40)
**LCX mean #**	79 (26–96)	81 (43–94)	71 (46–92)	29 (9–39)	35 (15–40)*	29 (21–39)

With anterior mantle field alone, overdosage to anterior portions of the heart was obvious (Table 1, Fig. 1). For instance, median maximal doses were 129% (up to 147% in individual patients) in the right ventricle, 123% (up to 142%) in the right atrium and 127% (up to 147%) in the right coronary artery. Compared with anterior mantle field alone, median maximal doses were significantly reduced to 117% in the right ventricle and 112% in the right coronary artery by combining the anterior mantle field technique with a monoaxial, bisegmental rotation boost technique (Table 1). Patients in whom the anterior mantle field was combined with a dorsal boost had significantly reduced median maximal doses the right atrium, 107%, and the right coronary artery, 109%.

When absolute doses in Gy were compared, the significant differences in dose to the right heart structures were lost, as patients treated with anterior mantle field alone received lower total mediastinal doses (mean 36.7 Gy) than patients in the MF+ROT (41.7 Gy) and MF+DORS (42.7 Gy) groups, indicating that the use of these boost techniques permitted dose increases in the mediastinum while maintaining the dose levels in the right heart structures seen with reduced-dose anterior mantle field alone. However, the use of a rotation boost technique led to significantly increased median absolute doses to the left atrium and the left circumflex artery, compared to mantle field alone. With the different techniques, median maximum doses to the right and left ventricle were between 48 and 52 Gy and between 44 and 46 Gy, respectively. In the coronary arteries, median maximal doses ranged between 46 and 50 Gy for the right coronary artery, 39 and 44 Gy in the left anterior descending artery and 34 and 42 Gy in the left circumflex artery.

To investigate the effect of shielding part of the heart with lead blocks in anterior mantle fields on the DVH results of cardiac structures (all patients and techniques considered together), we first quantified the percentage of 2-D heart area on the portal film shielded by lead blocks. The mean (± SD) percentage of the heart contour shielded by blocks was 36.3 ± 8.9% (range 18.2–53%). This percentage correlated inversely with several DVH parameters, e. g. the mean doses to both ventricles (Table 1), indicating sparing of the respective structures. All significant correlations applied equally to relative doses (in %) and absolute total doses (in Gy).

## Discussion

Irradiation of the mediastinum with anterior mantle field techniques, alone or followed by boost techniques, represents an outdated treatment technique that was, however, state of the art in the 1970s. The mantle field, defined as "a single anteroposterior radiation therapy field designed to treat in continuity the major lymph node-bearing areas above the diaphragm while maximally shielding the lungs in patients with lymphoma" [10] was first introduced by Kaplan in 1956 [5] and replaced the irradiation of individual nodal sites with separate fields. Extended-field radiotherapy of Hodgkin's lymphoma with megavoltage equipment, using e. g. the mantle field, was introduced in centers in Germany in the early to mid-1970s [11,12]. Concerns about the dose to the spinal cord and technical reasons led several centers to use anterior mantle fields alone or anteriorly weighted opposing fields rather than equally weighted opposing fields for mantle treatment [13]. The limited source-to-isocenter distance of cobalt machines and the necessity of large irradiation fields required extended source-to-skin distances which were achieved by positioning the patient on the floor in the supine position and placing blocks on a block holder above the patient. Introducing a dorsal mantle field would have required changing the patient position to prone and introduced unwanted dosimetric uncertainties. In cases where underdosage to the posterior mediastinum with an anterior mantle field was a concern, lateral oblique, dorsal or rotation boost techniques were employed [13]. Boost treatments could be performed on the treatment table, as fields were shorter due to limited treated mediastinal volumes. Although most reports focussed on cardiac toxicity after treatment with opposing photon beams, anterior mantle field technique in particular has been associated with high rates of late toxicity such as constrictive pericarditis [14-16].

Apart from a historic interest, a 3-D reconstruction of dose distributions achieved with such techniques is relevant because patients thus treated may require special cardiological attention. In the present study, 39% of the initial study cohort are alive today. Thus, detailed knowledge of the individual cardiac dose distribution in long-term survivors of mediastinal radiotherapy may help to identify patients at risk and aid the cardiologist in the interpretation of the patients' complaints and findings. Besides, the correlation of the reconstructed formerly applied 3-D dose distribution with the corresponding cardiovascular findings as obtained with non-invasive cardiac imaging techniques (e. g. cardiovascular magnetic resonance imaging [17]), may expand our knowledge of dose-effect relationships of cardiac structures such as the myocardium, coronary arteries or heart valves. It should be noted that solely studying the long-term survivors could potentially introduce a bias regarding the cardiac dose distribution in the overall cohort of n = 143 patients treated in the study period. The causes of death of the n = 88 non-survivors are not known and it can not be excluded that the cardiac doses differed between survivors and non-survivors. However, in preparation of a clinical investigation of long-term survivors, the dose reconstruction now presented was limited to this subgroup.

Although the cardiac effects of thoracic radiotherapy have been extensively studied in the 1980s and 1990s [3,4], direct comparisons between the dose applied to specific structures of the heart and consecutive patterns of cardiovascular structural and functional abnormalities are scarce in the literature. In a report on 144 survivors of mediastinal radiotherapy for Hodgkin's lymphoma, Glanzmann et al. calculated the total doses to the "anterior heart region" to be between 30 and 42 Gy in 93% of patients [3]. While the relative risk for myocardial infarction and for infarction or sudden death was 4.2 and 6.7, respectively, and valvular thickening was observed in 60% of patients after 30 years of follow-up, patients without additional cardiac risk factors (smoking, hypertension, hypercholesterinemia) were found to have no increased risk of cardiac events. In a recent systematic evaluation of Hodgkin's disease survivors 14 years (median) after chest radiotherapy, 42% had significant valvular defects, 75% conduction defects, and 30% a reduced peak oxygen uptake, a predictor of mortality in heart failure [1]. Further, a review of radiation-associated cardiovascular disease by Adams et al. suggests the following dose levels ("total cumulative radiation exposure to the chest") as a guideline for the selection of patients to be screened for cardiovascular disease [9]: pericarditis above 35 Gy, cardiomyopathy above 35 Gy (or above 25 Gy if anthracyclines were used), coronary artery disease above 30 Gy, valvular disease above 40 Gy. These data indicate that it may be of importance to reconstruct the local dose distribution and especially the localization of dose peaks if the total prescription dose was in the range of 30 to 40 Gy, in such patients in order to estimate the patients' individual cardiovascular risk, facilitating timely cardiovascular diagnostic procedures and adequate treatment before the occurrence of complications.

Cardiac structures now chosen for contouring and DVH analysis were limited by their visibility on standard non-enhanced planning CT scans. The heart cavities were contoured due to practicality although one could argue that the target tissue is the myocardium and not the heart content. Such considerations are unresolved until today in other body regions, e. g. concerning contouring of the whole rectum vs. the rectal surface in pelvic radiotherapy. Despite poor visibility, we contoured the major coronary arteries as we expected these to be major target organs mediating radiation-induced cardiac toxiticity, hypothesizing that with the anterior mantle technique, coronary arteries would differ in total dose resulting in specific patterns of coronary artery disease, as suggested in the older literature [18].

In the 55 long-term survivors now analyzed, anterior mantle-field technique alone was associated with a median maximal dose to the right ventricle of 128% and to the right coronary artery of 127%, with even higher doses in individual patients. Obviously, delivering part of the radiation dose via other techniques with lateral or dorsal beam entry reduced this relative overdosage in anterior portions of the heart. In patients treated with anterior mantle field followed by rotation or dorsal boost, significantly higher total doses could be delivered than with mantle field alone, without further increases in dose to anterior heart structures. This was achieved at the expense of irradiating parts of the left atrium which were either blocked or outside the high-dose region in the mantle-field technique. It is currently unclear if patterns of radiation-associated coronary artery disease differ from those seen in unirradiated patients. An older angiographic study in 15 patients with coronary artery disease after thoracic radiotherapy for different tumor entities (nine Hodgkin's lymphomas) found predominant left main and right ostial coronary artery disease [18]. Although the left main artery was not analyzed in the present study due to its short presentation on CT, high doses to the anterior heart, as resulting from anterior mantle field technique, could explain these locations.

Despite these technical efforts, the median maximal total doses to the right heart and right coronary artery were 48 Gy and 46 Gy, respectively. These structures also received the highest maximal doses in individual patients (58 Gy and 56 Gy, respectively). With a more modern opposed-field technique using a linear accelerator, in comparison, the overdosage to anterior heart structures should not be more than 6% of a manually calculated dose [19]. The adoption of involved-field radiotherapy with doses not higher than 30 Gy in favorable and intermediate Hodgkin's disease [7], e.g. in the current and previous generations of trials in Germany, has therefore reduced the maximum dose to any cardiac structure to slightly over 30 Gy. While the combination with chemotherapy may provide additional cardiotoxic effects, patients without mediastinal involvement, who previously would have been treated with an extended field in the form of a mantle field, would receive no dose to the heart in involved-field radiotherapy.

The 2-D percentage of shielded heart area correlated inversely with several DVH parameters. Especially the left and right ventricle as well as the branches of the left coronary artery benefitted from blocking. In structures that were partially blocked in most patients, such as left and right ventricle, mean and median dose but not minimum or maximum dose were correlated with the blocked heart area. The dose to the left and right atrium as well as the right coronary artery was not associated with heart shielding, suggesting that these structures are exposed to high radiation doses even with extensive heart shielding. This knowledge may be of interest even in the days of lower-dose involved-field radiotherapy when the position of coronary arteries is not usually considered.

The present analysis is limited by the lack of individual 3-D imaging studies in these patients. Variations in cardiac anatomy, e. g. branching of coronary arteries, could not be considered in the present design. The current data do not provide precise quantitative data on dose distribution in individual patients. However, for the whole cohort, the calculated doses should be a very good approximation. As part of an ongoing interdisciplinary study between cardiologists, radiologists and radiation oncologists, Hodgkin's disease survivors will be invited for extensive cardiologic tests including cardiac MRI [17]. As previously suggested [20], this project opens up the possibility to reconstruct the radiation dose in the individual patient's 3-D data set and directly correlate this with individual cardiac pathology. Even this approach, although considering individual anatomy, will not be able to take into account changes in cardiac morphology between the time of treatment (about 25 years ago) and clinical reevaluation.

## Conclusion

Our investigation documented the 3-D dose distribution of patients treated with anterior mantle-field technique 25 years ago. This technique, in some patients followed by mediastinal boost irradiation from non-anterior directions, was associated with dose peaks in anterior portions of the heart, especially the right ventricle and right coronary artery. Given the relatively high total radiation doses prescribed in this treatment era, our data set should provide a solid base for a detailed analysis of cardiac dose-effects in long-term Hodgkin's disease survivors.

## Methods

### Patients

Between 1978 and 1985, 143 patients were treated with mediastinal radiotherapy for Hodgkin's disease at the University of Würzburg. Of these, n = 55 (38.5%) were alive at the time of analysis in March of 2003 and were included in the present analysis, as these patients are potentially available for future cardiologic evaluation. Mean ± SD age at the time of treatment was 25 ± 10 years (range 6 to 49 years). A review of patient charts yielded the patient characteristics shown in Table 2.

**Table 2:** Patient Characteristics of n = 55 living patients treated with mediastinal radiotherapy for Hodgkin's disease between 1978 and 1985.

**characteristics**		**n (%)**
**sex**	male	29 (53%)
	female	26 (47%)
		
**stage**	I	13 (24%)
	II	28 (51%)
	III	14 (25%)
		
**B symptoms**		16 (29%)
**risk factors**	a	0 (0%)
	b	1 (2%)
	c	13 (24%)
	d	10 (18%)
		
**histology**	lymphocyte-predominant	7 (13%)
	nodular sclerosing	36 (65%)
	mixed	10 (18%)
	not available	2 (4%)
		
**involved regions**	cervical	27 (49%)
	supra-/infraclavicular	31 (56.4%)
	axilla	13 (23,7%)
	mediastinum	30 (54.5%)
	paraaortic	2 (4%)
	inguinal	3 (5%)
	spleen	11 (20%)
		
**treatment**	radiotherapy alone	33 (60%)
	chemoradiation	22 (40%)

### Dose reconstruction

For 2-D evaluation of the portal films of mantle field irradiation, heart borders were contoured and films scanned. Images were quanititatively analyzed using image processing software Scion Image vs. 4.0.2 (Scion Co., Frederick/MA, USA). The 2-D heart area was measured as well as the portion of the heart shielded by lead blocks.

For 3-D dose reconstruction, each treated patient was first assigned to one of four test patients (treated more recently for Hodgkin's disease) most closely resembling the anatomy of the treated patient, in particular with regard to heart shape. In each of the test patients, for whom planning CT studies in 1-cm slice thickness and spacing were available, the following structures were contoured in Helax TMS (Nucletron, Veenendal, Netherlands) treatment planning system on the basis of a digital atlas of thoracic CT anatomy [21] and of recent publications on cross-sectional heart anatomy [22,23]: left atrium, left ventricle, right atrium, right ventricle, left anterior descending (LAD) artery, left circumflex (LCX) artery, right coronary artery (RCA).

**Figure 2:**
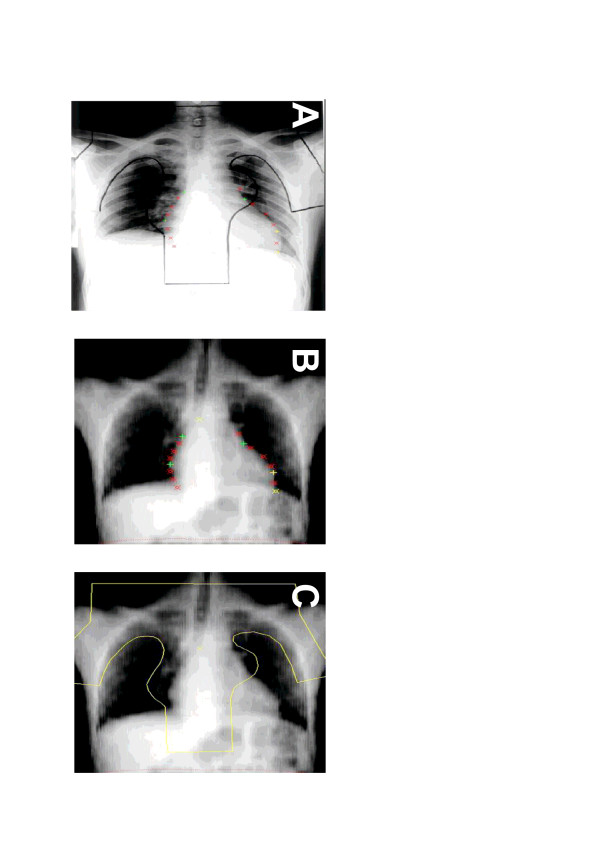
Matching procedure: Block contours were transferred from the mantle-field portal film of the treated patient (A) to the 0-degree digital radiographic reconstruction (DRR) of the CT data set of the test patient (C). This was achieved by matching the portal film to the DRR based on corresponding points of the heart contour (A and B). The quality of the match, with regard to the heart, is illustrated by the agreement of test points at the heart outline in A and B.

For reconstruction of the cardiac dose distribution, the original mantle-field portal film of the treated patient was matched to a digital radiographic reconstruction from a 0 degree angle of the test patient (Fig. 2). This matching procedure was based on specific corresponding landmarks on the heart outline of the treated patient and test patient, resulting in good matching results with regard to the heart, sometimes at the expense of incorrect matching concerning the lungs or bony structures, which were not part of the current analysis. All treatment details (source-skin distance, depth of prescription point, surface dose, dose at prescription dose, field size) were then entered into the planning system. All original treatments were performed with a cobalt-60 unit. Mantle-field irradiation was performed with patients positioned on the floor in supine position at typical source-skin distances of 120 cm. To reconstruct techniques other than mantle-field (rotation or dorsal boost techniques), treatment plans were reconstructed in a similar fashion, using simulation films documenting the anatomic isocenter position.

### Statistics

For each patient, all plans contributing to the mediastinal dose were added according to the total dose in Gy to which they were used. Different fractionation schemes were not corrected for. Cumulative dose-volume histograms for cardiac substructures were generated both for absolute doses (in Gy) and for relative doses (in %). DVH data were compared between groups treated with different techniques (mantle-field alone vs. mantle-field followed by different boost techniques) using the Kruskal-Wallis test with Dunn's post-hoc test (p < 0.05 considered significant). The correlation between 2-D area of heart blocked and 3-D DVH parameters was tested using the Spearman rank test.

## Competing interests

The author(s) declare that they have no competing interests.

## Authors' contributions

DV designed the analysis, reviewed patient data, performed dose reconstruction and statistical analysis and drafted the manuscript.

IS reviewed patient data, performed dose reconstruction and statistical analysis and revised the manuscript.

FS performed dose reconstruction and revised the manuscript.

OK performed dose reconstruction and revised the manuscript.

MK participated in the study design and revised the manuscript.

CA participated in the study design and revised the manuscript.

MF conceived of the study, participated in the study design and revised the manuscpript.
